# Targeting Akt in Hepatocellular Carcinoma and Its Tumor Microenvironment

**DOI:** 10.3390/ijms22041794

**Published:** 2021-02-11

**Authors:** Mariam Mroweh, Gaël Roth, Thomas Decaens, Patrice N. Marche, Hervé Lerat, Zuzana Macek Jílková

**Affiliations:** 1 Institute for Advanced Biosciences, Research Center Inserm U 1209/CNRS 5309, 38700 La Tronche, France; mariam.mroweh@univ-grenoble-alpes.fr (M.M.); groth@chu-grenoble.fr (G.R.); tdecaens@chu-grenoble.fr (T.D.); patrice.marche@univ-grenoble-alpes.fr (P.N.M.); herve.lerat@univ-grenoble-alpes.fr (H.L.); 2 Université Grenoble-Alpes, 38000 Grenoble, France; 3 Laboratory of Cancer Biology and Molecular Immunology, Faculty of Sciences I, Lebanese University, Hadath Beirut 6573-14, Lebanon; 4 Service D’hépato-Gastroentérologie, Pôle Digidune, CHU Grenoble Alpes, 38700 La Tronche, France

**Keywords:** AKT, HCC, tumor microenvironment, immune cells

## Abstract

Hepatocellular carcinoma (HCC) is one of the most common causes of cancer-related deaths worldwide, and its incidence is rising. HCC develops almost exclusively on the background of chronic liver inflammation, which can be caused by chronic alcohol consumption, viral hepatitis, or an unhealthy diet. The key role of chronic inflammation in the process of hepatocarcinogenesis, including in the deregulation of innate and adaptive immune responses, has been demonstrated. The inhibition of Akt (also known as Protein Kinase B) directly affects cancer cells, but this therapeutic strategy also exhibits indirect anti-tumor activity mediated by the modulation of the tumor microenvironment, as demonstrated by using Akt inhibitors AZD5363, MK-2206, or ARQ 092. Moreover, the isoforms of Akt converge and diverge in their designated roles, but the currently available Akt inhibitors fail to display an isoform specificity. Thus, selective Akt inhibition needs to be better explored in the context of HCC and its possible combination with immunotherapy. This review presents a compact overview of the current knowledge concerning the role of Akt in HCC and the effect of Akt inhibition on the HCC and liver tumor microenvironment.

## 1. Introduction

Hepatocellular carcinoma (HCC) is the most common type of liver malignancy (75–85%), and it ranks fourth among the causes of cancer-related deaths worldwide [1]. HCC generally emerges from a chronic inflammatory environment caused by various reasons. They could be of viral origins, like hepatitis C and B viruses (HCV, HBV), or caused by metabolic disorders leading to non-alcoholic fatty liver diseases (NAFLD) and non-alcoholic steatohepatitis (NASH). Moreover, chronic consumption of alcohol or consumption of toxins (such as aflatoxins) and hereditary diseases such as hemochromatosis lead to chronic liver inflammation, which could further develop into HCC [2]. 

Chronic liver inflammation often leads to fibrosis followed by cirrhosis and finally HCC. The changes in the state of the liver throughout the development of HCC are accompanied by a change in the tumor microenvironment (TME) profile, which sustains a niche favoring malignancy. The modulations in the status of TME affect an array of cells that include immune cells (resident and migratory), endothelial cells, hepatic stellate cells, and others. This leads to the differentiation of cells into those that support tumor development and progression: tumor-associated macrophages (TAMs), tumor-associated neutrophils (TANs), and cancer-associated fibroblasts (CAFs) [3]. The changes in the phenotypic and secretory profile of cells of TME result from the change in the transcriptome and/or an altered protein function in the cells accompanied by dysregulation of the complex signaling pathways in the cells. The alteration of the signaling pathways is common in HCC and is crucial for the progression of the tumor. RAS/RAF/MEK/ERK, HGF/MET, VEGF, PDGF, EGF, IGF, JAK/STAT, p53, MAPK, Wnt/β-catenin, TGF-β, and PI3K/Akt/mTOR [4] are among the altered signaling pathways.

HCC is challenging to diagnose and has limited therapeutic options. HCC patients often remain asymptomatic until they reach an advanced stage, hindering diagnosis. Alpha fetoprotein, the most widely used biomarker for HCC surveillance and diagnosis, is ineffective in accurately detecting early HCC [5,6]. Several advances have been made recently in the field of liver imaging and the development of novel biomarkers to attempt early detection of HCC, but most detected HCC cases are still diagnosed in advanced stages.

At an early stage of the disease, HCC can be treated by surgical resection, percutaneous ablation, or liver transplantation. At a later stage, the therapeutic options have been limited during the last decade to Sorafenib (a multikinase inhibitor) [7,8]. Recently, other first-line treatments such as lenvatinib and second-line treatments such as regorafenib and cabozantinib have been proposed for treatment. However, these drugs demonstrate no superior efficacy compared to Sorafenib [9]. In 2020, immunotherapy re-shuffled the cards with the combination Atezolizumab (an anti-programmed death-ligand 1 (PDL-1) antibody) plus Bevacizumab (an anti-vascular endothelial growth factor (VEGF) antibody), considerably increasing tumor response and survival outcomes and becoming the new first line therapy of advanced HCC. [10]. Nevertheless, only a minority of HCC patients benefit from this therapy, and alternative strategies are needed to augment host immune response [11].

**Figure 1:**
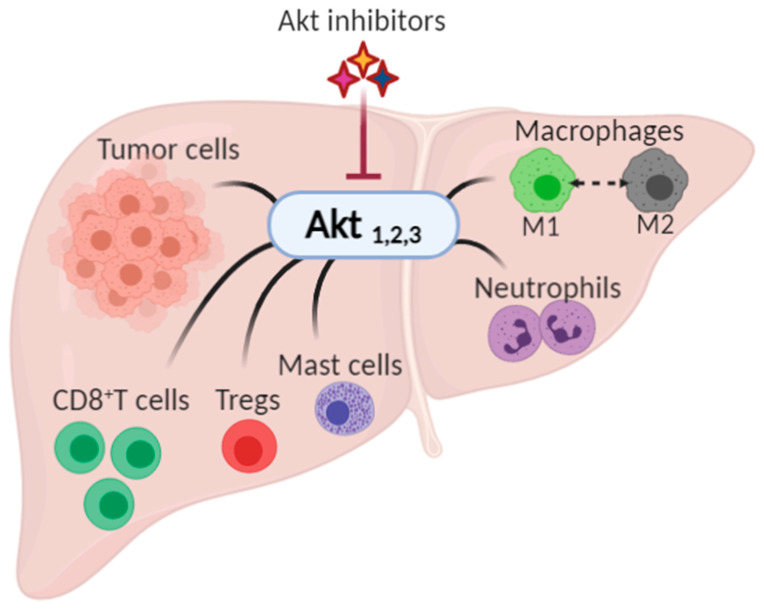
The possible impact of Akt inhibition on hepatocellular carcinoma and its tumor microenvironment. Akt, expressed as three isoforms, Akt1, Akt2, and Akt3, has been shown to play a role in the progression of cancer by controlling the growth, proliferation, and survival in tumor cells, and by modulating tumor microenvironment (namely CD8+ T cells, regulatory T cells (Tregs), mast cells, neutrophils, and macrophages).

As the search for therapies for HCC continues, several researchers are trying to pinpoint specific effector proteins that could be targeted. In this review, we demonstrate the role played by Akt (also known as Protein Kinase B) in the progression of HCC at the level of the tumor and TME and the growing interest in targeting Akt as a therapeutic option for HCC, Figure 1.

## 2. Akt Isoforms: Differences and Uniqueness

Akt is a serine/threonine protein kinase family member discovered in 1991 [12]. The ~56 kDa protein exists in mammals in three isoforms translated from three distinct genes: Akt1 (PKB alpha), Akt2 (PKB beta), and Akt 3 (PKB gamma). While the first two isoforms are constitutively expressed throughout the body—with a preference for insulin-sensitive tissues for Akt2—Akt3 is said to be expressed in the brain and the testes. On the structural level, Akt consists of the following three domains: (1) the amino-terminal pleckstrin homology (PH) domain, (2) a central domain sharing homology with other cAMP-dependent protein kinases (AGC kinases), and (3) a carboxyl-terminal domain serving as a regulatory domain. These three isoforms share homologies in their catalytic domains, but they diverge in the PH and regulatory domains [13]. Akt is said to be a master regulator serving as the center point in the phosphatidyl inositol 3 kinase (PI3K)/Akt pathway, which regulates several cellular processes encompassing cell survival, cell size/growth, survival, proliferation, glucose metabolism, transcription, protein synthesis, genome stability, and neovascularization, and thus any disturbance in this pathway has drastic effects on the cellular homeostasis [13]. The recruitment of various Akt isoforms to the plasma membrane is crucial for their activation. The PH domain binds to the phosphatidyl inositol-3,4,5-triphosphate (PIP3) generated by the action of PI3K at the plasma membrane. While there, Akt is prone to two phosphorylation events at the following threonine (Thr) and serine (Ser) residues: Thr 308 and Ser 473 for Akt1, Thr 309 and Ser 474 for Akt2, and Thr 305 and Ser 472 for Akt3. The Thr phosphorylation events are executed by phosphoinositide-dependent kinase-1 (PDK-1), whereas Ser phosphorylation events are executed by other kinases like the mammalian target of rapamycin complex 2 (mTORC2) and integrin-linked kinases (ILK). These two phosphorylation events are essential for Akt to attain its full function. However, it remains functional only with the Thr phosphorylation. In some instances, the activation of Akt surpasses the PIP3 checkpoint and can be activated by actin, heat shock protein (Hsp) 90, Hsp27, and Posh [13,14]. Various substrates are prone to phosphorylation by Akt, and they are said to be the downstream effectors of this pathway to regulate the various cellular processes. Some of these that can be highlighted are the following: proline-rich Akt substrate of 40 kDa (PRAS40), cyclin-dependent kinase (CDK) inhibitors P21 and P27, paladin and vimentin, inhibitors of KappaB Kinase alpha (IKKα), and tumor progression locus 2 (Tpl2). Moreover, Akt-mediated phosphorylation of tuberous sclerosis (TSC)1/2 complex and mTORC1 regulate cell growth. Survival has also been found among the cellular processes controlled by Akt through the direct inhibition of pro-apoptotic proteins like Bad or the inhibition of pro-apoptotic signals fired by the transcription factors such as Forkhead box protein O1 (FoxO1) [14]. Metabolism-related proteins like glycogen synthase kinase (GSK) 3β are also among the substrates for Akt. Although the different isoforms show an overlap in their activity, the substrate-specificities of Akt1/2/3 exist. These are dependent on the distribution of the Akt isoforms in the tissues, the differential activation of Akt by external stimuli (amplitude and timing of activation), the preferential intrinsic catalytic activity of the different isoforms, and the specific cell-context factors (subcellular localization and substrate proximity) [15]. Finally, a negative feedback loop exists for turning off this pathway, and this feedback is mediated by protein phosphatase 2A (PP2A), PH domain leucine-rich-repeat-containing protein phosphatase 1/2 (PHLPP2), and phosphatase and tensin homolog (PTEN) [13].

## 3. Akt in the Development and Progression of Hepatocellular Carcinoma

The PI3K/Akt signaling pathway has been receiving a lot of attention in cancer research as it has been shown to be hyper-activated in different types of cancer. The PI3K/Akt hyper-activation appears often due to the activating mutations in the effector molecules upstream and downstream of Akt rather than in Akt itself—except for the E17K mutation in the PH domain of Akt. The common activating mutations include the following: (i) a mutation hitting the catalytic subunit of PI3K rendering it constitutively active, (ii) loss of PTEN (whose role is to deactivate Akt), (iii) activation mutations of RAS and growth factor receptors, and (iv) gene amplification mutations of Akt and/or its effectors [16].

Activation of the Akt signaling pathway is closely related to the occurrence and development of liver inflammation and fibrosis [17,18,19] and is associated with a poor prognosis for HCC patients [20]. Importantly, a bioinformatic study analyzed 331 candidates for HCC prognostic factors among which all the three Akt isoforms were selected for further clinical validation, and the results showed a correlation between tumor aggressiveness and poor prognosis [21].

The contribution of the various Akt isoforms in the progression of HCC has been explored by research carried out over the years. As far as Akt1 is concerned, a study in 2019 showed that the Akt1-mediated phosphorylation of mTORC2 is crucial for triggering hepatocarcinogenesis in humans and mice, as it contributes to cellular growth through c-Myc activation [22]. Further, the altered metabolic state of the liver in HCC commonly exhibits a marked up-regulation of aldose reductase. Interestingly, aldose reductase has been shown to interact with the catalytic domain of Akt1, leading to an activation of the Akt/mTOR pathway [23]. Moreover, the TME components can lead to an activation of Akt. For instance, an in vitro study mimicking the augmented TME polyamine levels in HCC showed a subsequent increase in the levels of Akt1 along with those of ornithine decarboxylase, spermidine/spermine N1 acetyl transferase, hypoxia inducing factor 1α, matrix metalloproteinase 9, VEGF, and downregulated p27. The previous fluctuations resulted in an increase in the proliferation and the migration of HepG2 cells (HCC cell line) [24]. To further support the role of Akt1 in HCC progression, a study demonstrated that the overexpression of myriostylated Akt1—and thus constitutively active Akt1—led to a liver tumor development in mice, and its combination with S-phase kinase-associated protein overexpression exacerbated the phenotype [25]. Interestingly, a real-time imaging study of Akt1 in HCC cells demonstrated that the nuclear translocation of Akt1 is independent of the phosphorylation events at the Thr308 and Ser473 [26]. In contrast, a study showed that a decrease in β-catenin levels in the HepG2 cell line showed no change in the Akt1 expression level, while it decreased the phosphorylated and consequently decreased cell growth. This stresses the cross-communication between the different signaling pathways activated by the signals from TME, which converge onto the Akt1 activity and its effect on HCC progression [27].

Akt2 has also been shown to be a contributor to HCC progression, and some researchers argue that its role in HCC prognosis surpasses that of Akt1. A study by Xu et al. detected overexpression of Akt2 in 38% of the HCC tissues of the studied cohort with a moderate or less expression of Akt1 in all the cases. The high expression of Akt2 correlated with the histopathological differentiation, portal invasion, and the number of tumor nodules, while Akt1 did not correlate with any of these clinicopathological features [28]. An in vitro study conducted to investigate the role of Akt2 in the proliferation and migration of HCC cells showed that Akt2 was regulated by STAT3. The ablation of STAT3 by small-interfering RNA (siRNA) led to a decrease in the phosphorylated form of Akt2 and a subsequent decrease in the proliferation and migration of HCC cells. Moreover, the in vivo transfection with siRNA against STAT3 decreased the pace of the tumor growth, a process that was reversed by the expression of Akt2. This points to the significant role of Akt2 in the growth of the tumor [29]. Another in vivo study highlighted the importance of Akt2 in the process of hepatic steatosis and carcinogenesis. In this study, the hydrodynamic transfection of a mutated form of PI3K subunit alpha alone into mouse livers led to hepatic steatosis, whereas the transfection with a combination of a mutated form of PI3K subunit alpha combined with either NRASV12 or c-Met in the mouse liver led to the development of tumor nodules, which exhibited an increase in the activation of Akt/mTOR and RAS/MAPK signaling pathways. The ablation of Akt2 in mouse livers inhibited both the hepatic steatosis in the former case and tumorigenesis in the latter one [30]. Interestingly, a study of Zebra fish showed that the induced expression of oncogenic Kras led to the development of HCC having great resemblance to the human tumors with an elevated Akt2 activation [31]. On the other hand, the concomitant and systemic deletion of Akt1 and Akt2 in adult mice caused hypoglycemia, liver inflammation, and death [32], pointing to potential toxicities of strong and long-lasting Akt inhibition.

Despite the previous knowledge about the focused expression of Akt3 in the testes and brain, some studies have shown the implication of Akt3 in HCC. A series of studies on micro-RNA (miRNA) profiles in HCC revealed the downregulation of miRNA-144, and miRNA-582-5p. The downregulation of the previously mentioned miRNAs resulted in a sustained expression of their downstream targets. Both the aforementioned miRNAs showed Akt3 among their targets, and their subsequent downregulation supported tumor progression and growth. This supports the contribution of Akt3 in HCC progression [33,34].

## 4. Akt Modulates the Immune Cells

In addition to direct anti-tumor activity, there is growing evidence that targeting the Akt pathway has an indirect anti-tumor activity that is mediated by the response of immune cells [35]. In fact, TME, which is generally immunosuppressive and metabolically stressed, can be modulated by Akt, as this pathway is essential to the differentiation, maturation, and functioning of many immune cells. At present, the immunomodulation by Akt is best characterized at the level of T cells and macrophages.

### 4.1. Akt Regulates the Functions and Fate of T Cells

The regulation of the nutrient metabolism in the T cells is important for the control of their differentiation, as it shapes their function and survival. Quiescent T cells require oxidative catabolic metabolism and use low amounts of nutrients. T cell activation through T cell receptors (TCR) induces a metabolic switch to aerobic glycolysis and anabolic metabolism to sustain cell division and differentiation. Then, different subsets of activated T cells adopt fine-tuning of metabolism homeostasis according to their functions and fate. For example, the glycolytic rates are higher in Th1, Th2, and Th17 than in the T regulatory cells (Tregs). Tregs use fatty acid oxidation for their energy demand, while memory T cells use mitochondrial oxidative phosphorylation and fatty acid oxidation for their long-term persistence [36].

The PI3K-Akt pathway orchestrates the nutrient uptake and utilization within the cell. Thus, it is a key pathway to regulate the functions and fate of T cells. The TCR and CD28 co-stimulation by their respective ligands is known to engage the PI3K/Akt/mTOR signaling cascade [37]. The differentiation into effector T cells and memory T cells is achieved, in part, by asymmetric division, where daughter cells contain different amounts of active mTORC1. Thus, the high levels of active mTORC1 in effector T cells increase glycolytic activity, and the low levels in memory T cells increase lipid metabolism. This event is achieved by RagC mediated translocation of mTOR to the lysosomes through a CD98 and leucine-dependent mechanism [38]. The mTOR inhibition in T cells induces tolerance through the T cell energy [39] and blocks the differentiation to effector T cells leading to the generation of FoxP3+, Tregs, and CD8+ memory T cells. This is associated with the lower activation of the transactivation factors STAT4, 6, and 3 in response to IL-12, 4, and 6 stimulations. Interestingly, and in contrast with hepatocytes, the Akt-dependent induction of mTORC1 activity in the T cells does not require mTORC2 for their differentiation into Th1 and Th17. In contrast, the T cell differentiation into Th2 requires functional mTORC2 [40]. TCR can induce the mTOR activation through upstream PI3K/Akt activation, which is the signaling that is enhanced by costimulatory receptors (e.g., CD28) [37].

In contrast, cytotoxic T-lymphocyte-associated protein 4 (CTLA-4) and PD-1 can antagonize the mTOR activity via Akt and PI3K inhibition. Indeed, the CTLA-4 mediated inhibition of Akt is dependent on PP2A. The PD-1-induced Akt inhibition involves a blockade of the CD28 cytoplasmic tail function (probably through SHP1/2), thus preventing the synthesis of PI3P by PI3K. In addition, the PD-1-induced PI3K/Akt pathway inhibition is more potent to block T cell differentiation than the CTLA-4-induced PI3K/Akt inhibition [41]. Notably, the PI3K/Akt/mTOR pathway also regulates lymphocyte trafficking through the modulation of CD62L and CCR-7 expression [42].

Regarding the Akt isoform dichotomization in T cell activation, recent publications suggest a divergence in Akt1, 2, or 3 functions. The Akt-1 isoform downregulates proliferation of the thymus-derived Tregs, thus facilitating antigen-specific Th1/Th17 responses. On the other hand, Akt2 increases the proliferation of Tregs and suppresses the antigen-specific Th1/Th17 responses. Furthermore, the treatment with a specific Akt1 inhibitor suppresses disease progression in a mouse model of autoimmune encephalomyelitis [43]. The Akt3 signaling in T cells, and not neurons, is necessary for maintaining the central nervous system integrity during an inflammatory demyelinating disease (in vivo model of myelin-oligodendrocyte glycoprotein-induced experimental autoimmune encephalomyelitis) [44]. Further studies are needed to clarify the role of each Akt isoform in the T cell activation process and antitumor activity.

In conclusion, the Akt pathway is at the epicenter of the T cell activation/differentiation process. This needs to be taken into consideration while choosing the therapeutic targeting of this pathway.

### 4.2. Macrophage Polarization

Macrophages are a heterogeneous population of cells arising from the myeloid lineage and are involved in innate immunity. The roles of these cells during pathogen encounters are broad, and they encompass antigen processing, presentation, orchestration of inflammatory response, clearance, and repair. A dichotomy between “classically activated macrophages” and “alternatively activated macrophages” depending on different stimuli has been described and led to the emergence of an “M1” versus “M2” terminology. As simple as this classification sounds, in reality, such clear division was challenged by the discovery of TAMs that do not fit in this classification system. Different subsets of M2 macrophages (M2a, M2b, M2c, and M2d) were brought into the spotlight in accordance with their transcription profiles and responses to the different stimuli. Importantly, the presence of TAMs is generally associated with a poor prognosis in solid tumors [45].

The different polarization states of the macrophages result in different modes of action according to the tissue environment based on the signals that they receive from the surrounding cells. M1 macrophages exhibit a pro-inflammatory function along with a microbicide activity and resistance to pathogens. M2 macrophages predominantly mount an anti-inflammatory response, parasite control, tissue remodeling, and immune modulation. The macrophage polarization fate is dictated by the regulation of cytokine production, phagocytosis, autophagy, apoptosis, and metabolism. All of these pathways seemed to have Akt as the converging node. In fact, signaling cascades controlled by PI3K-Akt largely contributed to macrophage polarization [46].

The PI3K/Akt pathway plays a key role in the increase of anti-inflammatory markers such as arginase-1 and IL-10 and inhibition of the production of pro-inflammatory cytokines [47,48]. It has been described that activation of the PI3K-Akt pathway results in increased polarization of M2 macrophages [46], and BMP7 and SMAD 1/5/8 might play a major role in these events [49]. Expectedly, published data also suggest that the inhibition of either PI3K or mTOR results in M1 macrophage polarization [50,51]. For example, rapamycin treatment has been found to promote M1 polarization [52]. Nevertheless, the Akt pathway can also be triggered in macrophages by TLR4 activation [53], activating downstream NFkB [54] and mTORC1 [55], which are responsible for M1 genes transcription.

While differences in the PI3K/Akt pathway involvement upon macrophage activation might arise from distinct tissue environments or macrophage origins between studies, the differences in the Akt isoforms (Akt 1, 2, or 3) play an important role in this matter [46]. Studies using double and triple gene knockout have showed increasing evidence that each Akt isoform possesses non-overlapping functions. Indeed, in a study using dextran sodium sulfate-induced colitis, the genetic ablation of Akt1 isoform exacerbated the disease; however, the ablation of Akt2 in these mice protected them. This difference was due to the M1 profile and M2 profile in these two cases, respectively [56]. Moreover, studies have shown that knocking down of the Akt1 expression promotes the upregulation of iNOS and IL-12β (M1 activation) and suppresses TLR4-induced M1 macrophage activation. In contrast, as reviewed previously, the knockout of Akt2 resulted in an M2 phenotype along with elevated M2 markers (Arg1, Ym1, and Fizz1), endotoxin tolerance, and elevated levels of IL-10 [46].

Still, the role of the Akt isoform subcellular localization in macrophages has been poorly documented, and a further level of complexity exists based on the acute versus chronic activation of Akt that remains to be tested in terms of macrophage fate. In conclusion, activation of macrophages is highly dependent on the Akt pathway, but the full picture of Akt involvement in macrophage polarization remains to be completed, especially concerning the role of each Akt isoform in TME of HCC.

### 4.3. Other Cells in TME

TANs are major players in the TME of HCC. They recruit both macrophages and Tregs to promote the progression of HCC [57]. Akt signaling cascade is involved in migration, degranulation, and O_2_ production, and the Akt2 isoform has a predominant role in regulating neutrophil functions [58].

Additionally, mast cells modulate the immune response and mediate angiogenesis. It has been demonstrated that the recruitment of mast cells increases angiogenesis by PI3K-Akt-GSK3β-AM signaling, which can be reversed by Akt inhibition [59].

## 5. Targeting Akt in the Management of HCC

Numerous compounds have been reported to inhibit the PI3K/Akt/mTOR pathway, as reviewed previously [60,61]. Akt inhibitors have been tested as an anti-tumor treatment in several preclinical studies and early clinical phase trials, mostly in gynecologic and prostate cancers. Furthermore, data from preclinical and clinical studies are also available for HCC (as summarized in Table 1).

Classical ATP-competitive Akt inhibitors such as ipatasertib (GDC-0068) and capivasertib (AZD5363) are currently in phase-I and phase-II clinical trials for mono- or combination-therapies. However, because the ATP-binding pocket of Akt is highly conserved among kinases, the selectivity of these inhibitors is limited, and their use is associated with side effects during treatment and lack of efficacy. The efforts to identify more specific and selective small molecules have resulted in the development of allosteric inhibitors such as MK-2206, ARQ092, and ARQ751 [62,63].

**Table 1:** Akt inhibitors in the management of HCC.

Inhibitor	Mechanism of Action, Structure	Experiment Setup	Antitumor Effect	Effect on TME	Clinical Trial
GDC-0068 [64,65,66]	ATP competitive AKT inhibitor 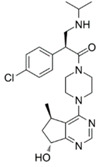	Combination of GDC-0068 with Sorafenib in HepG2 and Huh7 sorafenib-resistant cell-lines	- synergistic antitumor effect - promotion of apoptosis - induction of - autophagic cell death	Not investigated	Phase-I/II including multiple solid tumors treated by GDC-0068 in monotherapy or in association with abiraterone + prednisolone: safe and tolerable in monotherapy or in combination
AZD5363 [67,68]	ATP competitive AKT inhibitor 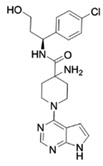	Single agent in HepG2 and Huh-7 HCC cells Combination with FH535 (β-catenin inhibitor) in THH, Hep3B and HepG2	- inhibition of tumor proliferation - induction of cell-cycle arrest - promotion of apoptosis - promotion of autophagy through p53 activation	Not investigated	Phase-I, AZD5363 in monotherapy, in multiple advanced solid tumors including liver cancer (NCT01895946): safe and tolerable, no data on antitumor response Phase-I including multiple solid tumors harboring AKT mutations (NCT02465060)
ARQ 092 [69,70]	allosteric pan-AKT inhibitor 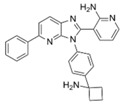	Single agent and in combination with Sorafenib in a DEN-induced cirrhotic rat model of HCC and in Hep3B, HepG2, Huh-7, PLC/PRF, and HR4 HCC cell lines	- inhibition of tumor proliferation - promotion of apoptosis - synergistic antitumor effect when combined with sorafenib	- anti-angiogenic effect - improved liver fibrosis - decrease of intrahepatic neutrophils and macrophages	No clinical trial
ARQ 751 [71]	allosteric pan-AKT inhibitor Chemical structure of ARQ 751 is currently unavailable.	Single agent and in combination with sorafinib in DEN-induced cirrhotic rat model of HCC	- inhibition of tumor proliferation - synergistic antitumor effect when combined with sorafenib	- Improved liver fibrosis	Phase-Ib (NCT02761694) in solid tumors with PIK3CA/AKT/PTEN mutations including HCC: ongoing
MK-2206 [72,73]	allosteric pan-AKT inhibitor 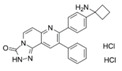	Single agent in Human Huh7, Hep3B, and HepG2 HCC cell lines	- Promotion of apoptosis - Inhibition of cell proliferation - Induction of cell cycle arrest	Not investigated	Phase-II trial, MK-2206 in monotherapy in advanced HCC previously treated (NCT01239355): discontinued due to discouraging results

First, an ATP-competitive Akt inhibitor GDC-0068 showed promising antitumor activity, ranging from tumor growth delay to regression in multiple tumor xenograft models as a single agent to the potentialized antitumor activity of classic chemotherapeutic agents [64]. A high basal level of phospho-Akt, PTEN loss, and PIK3CA kinase domain mutations were predictive of a better response to GDC-0068 [64]. More specifically, in an in vitro model of sorafenib-resistant HCC cells, the exposure to GDC-0068 combined with sorafenib restored sensitivity to Sorafenib by switching autophagy, one of the known resistance mechanisms, from a cytoprotective role to a death-promoting mechanism. This association induced a synergistic antitumor effect suggesting, at the time, that GDC-0068 represents a good candidate for further clinical trials in combination with Sorafenib [65]. In clinics, to this day, GDC-0068 was tested in a phase-I basket trial with multiple solid tumors including only one HCC patient without any further study carried out in this pathology [66]. This compound is currently mostly studied in prostate and breast cancers in combination with other anticancer agents.

AZD5363, also known as Capivasertib, is another Akt inhibitor that binds to and inhibits all Akt isoforms. In a large number of cancer cell lines, it has been shown to decrease FOXO3a phosphorylation through Akt inhibition, leading to FOXO3a translocation to the nucleus where it can “switch on” the expression of genes, such as p27, FasL, and BIM by inducing cell-cycle arrest and promoting apoptosis [74]. Moreover, AZD5363 suppresses the proliferation of human HCC cell lines, HepG2 and Huh-7, by inhibiting the phosphorylation of downstream molecules in the Akt signal pathway in a dose- and time-dependent manner [67]. AZD5363 was studied in combination with the β-catenin inhibitor (FH535) in vitro. This combination induced a stronger effect on cell death and displayed antiproliferative effects on transformed human hepatocytes through inhibition of cell-cycle progression, enhanced autophagy marker protein expression, and autophagy-associated death. These promising results suggest that inhibiting both Akt and β-catenin pathways may represent a new therapeutic way of treating HCC that would, however, require further preclinical and clinical investigations [75]. AZD5363 was tested in a phase-I trial in multiple advanced solid tumors including HCC, and it showed acceptable safety and tolerability profiles [68]. Moreover, it is still under investigation in a large phase-I study, the MATCH screening trial, that includes multiple solid tumors harboring druggable mutations including Akt mutations (NCT02465060).

ARQ 092 is a small allosteric pan-Akt inhibitor that showed interesting results in a diethylnitrosamine- (DEN) induced rat model of HCC developing on cirrhosis, which was assessed by MRI. The ARQ 092 treatment induced downregulation of the mTOR pathway with a decrease in the active phosphorylated form of Akt and its downstream actors. ARQ 092 improved the tumor response, normalized the vascularization, and significantly decreased the fibrosis of the surrounding liver [69]. A combination of ARQ 092 with Sorafenib synergistically decreased the tumor progression with the promotion of apoptosis and reduction of tumor proliferation and angiogenesis [70], and similar effects were observed when ARQ 751, a next-generation allosteric pan-Akt inhibitor, was used [71]. ARQ 751 is now tested in clinics, in a phase-Ib basket trial, as a single agent or in combination with other anticancer drugs in the case of solid tumors with PIK3CA/Akt/PTEN mutations including HCC (NCT02761694).

MK-2206, another allosteric Akt inhibitor that has been studied in vitro and in vivo in many cancers, has shown interesting in vitro activity resulting in cell-cycle arrest, inhibition of cancer cell proliferation, and promotion of apoptosis in human HCC cell lines [72]. It was, later, studied in a phase-II trial including patients with advanced HCC that was previously treated but was prematurely arrested due to discouraging results (NCT01239355). Another phase-II trial in the case of advanced biliary cancer was also stopped after eight inclusions due to the absence of clinical efficacy as a single agent but with an acceptable safety profile (NCT01425879) [73]. Further trials are needed on this front in combination with other targeted therapies.

The data concerning Akt inhibitors in HCC are still preliminary and future clinical development may have to involve combinations with other targeted therapies such as β-catenin inhibitors or immune checkpoint inhibitors to improve the care of HCC patients.

## 6. Conclusions and Future Perspectives

Although the Akt inhibitors have been intensively studied during the past decade in the context of cancer, their effect on TME has received less attention until very recently. Interestingly, recent studies suggest that apart from their direct anti-tumor activity, the Akt inhibitors have the capacity to modulate TME and to switch it from pro-tumoral to anti-tumoral. The inhibition by AZD8835 in pre-clinical mouse tumor models directly increased CD8^+^ T-cell activation, while Tregs, macrophages, and myeloid-derived suppressor cells were strongly suppressed [76]. Similarly, in several different tumor-bearing mice, the Akt pathway inhibitor, MK-2206, caused the selective depletion of suppressive Tregs, which was associated with enhanced cytotoxic CD8 responses [77]. The Akt inhibitor, AZD5363, was administered as an adjuvant after radiotherapy in tumor-bearing mice which was associated with marked reductions in tumor vascular density, a decrease in the influx of CD11b+ myeloid cells, and a failure of tumor regrowth [78]. Similarly, in the rat model of HCC, which accurately recapitulated the scenario and TME of human HCC [79], the Akt inhibition by ARQ 092 was associated with the modulations of TME mainly in the form of a decrease in the accumulation of intrahepatic neutrophils and macrophages [70]. Recent studies have also demonstrated the effect of the Akt inhibitors on TME in cancer patients. For instance, in HER2 negative breast cancer patients, two oral doses of the Akt inhibitor, MK-2206, were associated with a favorable immune profile in TME, including increased CD3^+^CD8^+^ density and greater expression of interferon genes [80]. However, there is no study yet assessing the impact of Akt inhibition on TME in HCC patients.

In conclusion, the Akt pathway plays an important role in the regulation of several processes in the development and progression of HCC. It does this by controlling the growth, proliferation, and survival in tumor cells, on one hand, and by modulating TME, on the other. TME was the underestimated player until recently, but its involvement in HCC progression is now well-recognized. In this article, we have emphasized the importance of considering the effects on TME while developing strategies inhibiting Akt in HCC. Targeting Akt can lead to a favorable change in the immune microenvironment and thus provides a rationale for combining these agents with immunotherapeutics. Additional studies are warranted to pave the way for combining Akt inhibition with immunotherapy in HCC. Furthermore, the isoforms of Akt converge and diverge in their designated roles, but the currently available Akt inhibitors fail to display an isoform specificity. Thus, additional investigations are needed to define the isoform specificity of Akt-related therapeutic targets to trigger a beneficial immune response in HCC patients.
